# Digital habitus and the sociotechnical transformation of everyday life in the United Arab Emirates: a hybrid framework of governmentality, surveillance capitalism, and Gulf state modernity

**DOI:** 10.3389/fsoc.2026.1766932

**Published:** 2026-04-13

**Authors:** Abdelrahim Abulbasher

**Affiliations:** Department of Social Sciences, University of Kalba, Sharjah, United Arab Emirates

**Keywords:** digital governance, digital habitus, digital sociology, Gulf modernity, identity, surveillance capitalism, UAE

## Abstract

**Introduction:**

Rapid digitalization in the United Arab Emirates (UAE) has reshaped everyday life, yet existing scholarship has not fully captured how digital practices are embedded within broader sociotechnical and political-economic transformations. In particular, there is a need for a framework that integrates cultural dispositions with structures of power, governance, and market logics.

**Objective:**

This paper develops a conceptual framework to explain how digital practices are produced, structured, and experienced in the UAE, with a focus on the transformation of everyday life.

**Methods:**

The study adopts a theoretical and interpretive approach, building on the concept of *digital habitus* derived from Pierre Bourdieu's theory of habitus. It synthesizes insights from governmentality (associated with Michel Foucault), surveillance capitalism (as articulated by Shoshana Zuboff), and scholarship on Gulf state modernity to construct an integrated analytical framework.

**Results:**

The paper proposes a hybrid framework that conceptualizes digital habitus as shaped by the interaction of state governance, corporate digital infrastructures, and culturally specific forms of modernity. It highlights how everyday digital practices in the UAE are simultaneously enabled and constrained by systems of surveillance, data extraction, and state-led modernization, producing distinct patterns of behavior, identity formation, and social interaction.

**Conclusion:**

By integrating cultural, political, and economic dimensions, the proposed framework advances understanding of sociotechnical transformation in the UAE and offers a foundation for future empirical research on digital life in non-Western contexts.

## 1 Introduction

The United Arab Emirates (UAE) has experienced a rapid and comprehensive digital transformation in the last decade, altering how its citizens and residents work, interact, express identity, and relate to public institutions. Over the past decade, digital transformation has become integral to national development, as seen in government initiatives, investments in infrastructure, and promotion of a knowledge-based economy (Alaleeli and Alnajjar, 2020; Alzarooni et al., 2024; Tariq, 2023; Ignatow and Robinson, 2017; Alfalah et al., 2025). Policies such as Smart Dubai, the UAE Artificial Intelligence Strategy, and digital governance frameworks have modernized institutions and reshaped daily practices. As a result, new forms of digital participation have emerged in specific cultural, governance, and economic contexts.

Despite extensive scholarly attention to digitalization in the Middle East and the Gulf, sociological theory has paid little attention to the social and cultural effects of digital transformation in the Arabian Gulf (Zayani and Khalil, 2024). Much of the existing literature examines the economic policies of countries undergoing digital transformation and how they use it to practice censorship and political control. Yet it offers little in terms of theoretical guidelines to help understand the hybrid nature of Gulf digital modernity. Specifically, it lacks a framework that can capture how state-led digital initiatives, private-sector data platforms, and culturally specific future visions interact to co-produce everyday routines, expectations, and modes of self-regulation.

This paper attempts to address this gap by introducing a conceptual framework grounded in digital habitus, adapting Bourdieu (1984) insights to the dynamics of digital environments. Digital habitus highlights how people internalize digital norms, practices, and expectations. In the UAE, digital adoption fits state narratives of progress. Unique local factors—state direction, commercial mediation, and Gulf norms—shape this transformation. Digital habitus offers a tool for examining social change in Gulf societies, where such analysis remains underdeveloped (Rahman and Al-Azm, 2023). The main argument of this paper is that digital habitus in the UAE is shaped by structural forces unique to the Gulf, requiring a framework beyond Western models. The article proposes a framework that integrates three theoretical strands (governmentality, surveillance capitalism, and Gulf state modernity) to capture the structural forces shaping everyday digital practices. Together, these approaches guide two questions: (1) How do governance, markets, and modernization shape digital orientations in the UAE? (2) How does digital habitus mediate identity, civic engagement, community, and social stratification?

The article proceeds as follows. The literature review discusses key scholarship on digital governance, surveillance capitalism, and Gulf state modernity. The framework section details the integrated model based on digital habitus. The discussion applies this model to examine identity, community, stratification, and civic participation. The conclusion synthesizes the argument and suggests avenues for further research in the Middle East.

## 2 Background and literature review

### 2.1 Digital transformation and state modernity in the Gulf

Gulf countries, especially the United Arab Emirates, have undertaken rapid modernization processes under state leadership, with digital technology central to national development agendas. Prominent UAE initiatives such as Smart Dubai and the AI Strategy exemplify this approach (Ahmat et al., 2024; Alzaabi et al., 2025; Alsuwaidi et al., 2025). These programs integrate digital tools into governance, healthcare, transport, and administration, positioning technology both as an instrument and symbol of Gulf modernity (Kanna, 2011). Unlike digital transformation in Western contexts, which is generally market-driven, in the Gulf, it is centralized and directed by the state (Ulrichsen, 2016; Sarker et al., 2023; Merhaj, 2021). States closely manage regulation and digital infrastructure, embedding platforms in citizens' daily routines. This model aligns with Gulf state modernity, in which digital technologies reinforce identities, maintain order, drive the economy, and advance critical infrastructure, such as educational systems and platforms (Davidson, 2019; Rahman and Al-Azm, 2023). These infrastructures offer opportunities and impose discipline, shaping public and private life.

### 2.2 Governmentality and digital governance

**Figure 1:**
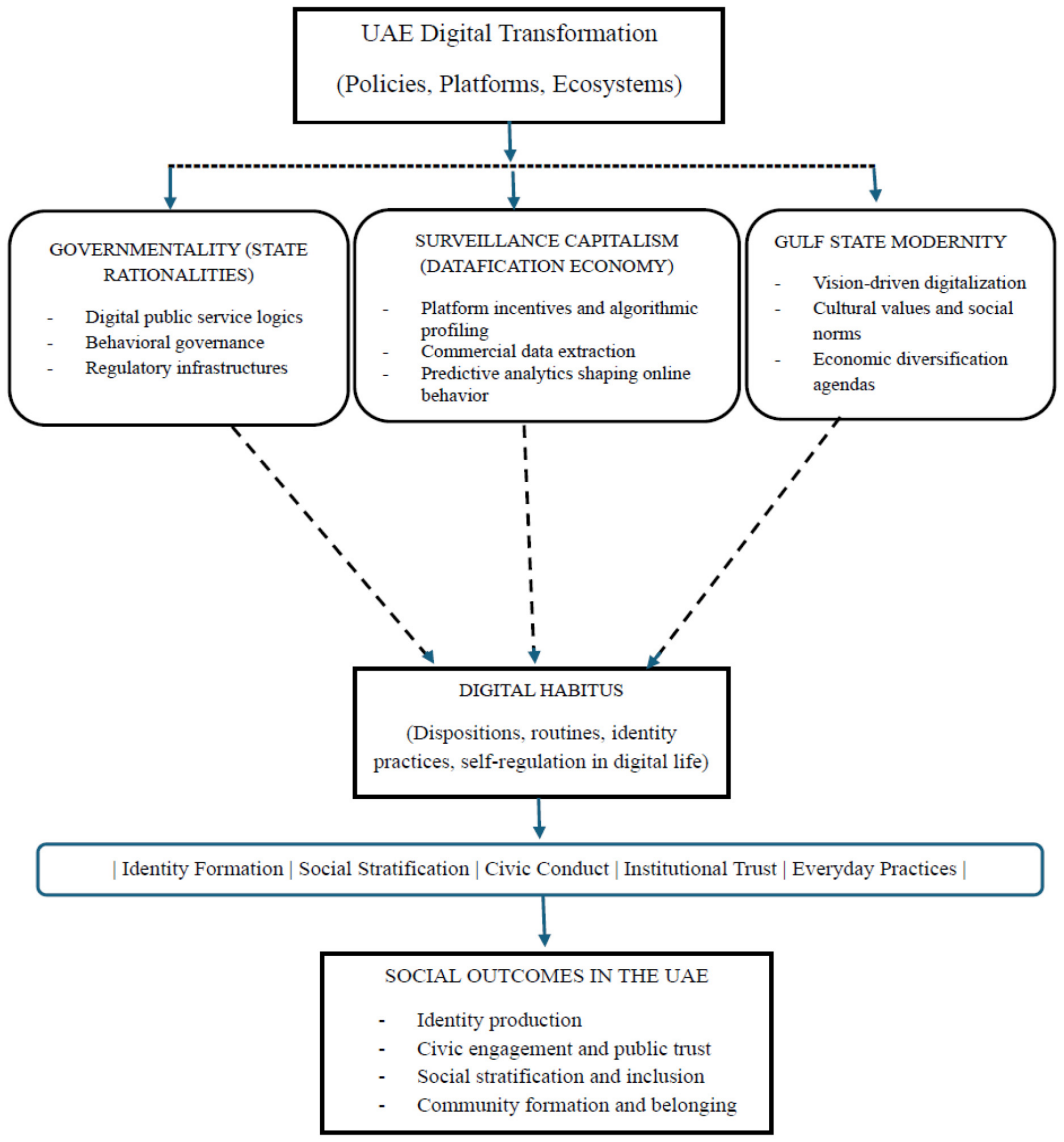
Digital habitus framework in the UAE: linking governance, market, and modernity to social outcomes.

Foucault's concept of governmentality examines how states exert power through regulation, norms, and expectations (Foucault, 1991; Lyon, 2007; Senellart and Ewald, 2009). Like many Gulf countries, where advanced digital infrastructures, including artificial intelligence, are used not only to provide digital platforms for government services and to influence the daily activities and behavior of citizens and residents, but also to remain resilient to security threats (Seloom, 2026). In the UAE, as shown in Figure 1, governmentality manifests in digital platforms that shape resident behavior. In the Gulf countries, advanced technologies are frequently used as governing technologies, helping to advance their economic and financial institutions, promote social and cultural development, and maintain national security by preventing crimes and monitoring illegal activities (Seloom, 2026; Bi et al., 2025). In the UAE, for instance, digital ID systems such as the UAE Pass standardize online transactions, enable e-government, and embed compliance into everyday life (Sarker et al., 2023). Such platforms align self-regulation and state objectives, resonating with Rose's (1999) concept of indirect power through norms and technology.

Government services in the UAE have become highly digitalized, combining traditional governance with technological innovation (Schou and Hjelholt, 2018). Individuals internalize norms of digital engagement, efficiency, and transparency, contributing to a digital habitus in which systematic behaviors are both socially reinforced and institutionally mandated (Ignatow and Robinson, 2017).

### 2.3 Surveillance capitalism and Gulf digital infrastructures

Digital transformation is widely used in many countries across the globe to achieve various outcomes, such as promoting and advancing economic and financial institutions and systems, monitoring and controlling citizens, and maintaining national security (Seloom, 2026; Weber, 2025; Codreanu, 2023; Rahman and Al-Azm, 2023; Zayani and Khalil, 2024; Perdana and Mokhtar, 2025). Its uses vary from one country to another and from region to region, including those of authoritarian and semi-authoritarian states. While some authoritarian countries primarily use digital transformation for surveillance and censorship, others incorporate social, political, and economic aspects into their digital transformation models, creating three types of systems based on their uses.

First, in Western countries, digital transformation is often used as a tool of market-driven surveillance capitalism (Eom and Lee, 2022; Durante, 2026). Second, although countries such as China, Iran, and Russia use digital information to promote economic development, they also widely use it to enhance censorship and reinforce authoritarian control, merging high-tech surveillance and legal enforcement (Weber, 2025; Codreanu, 2023; He, 2024). The third model is reflected in states such as Singapore, Kazakhstan, Kyrgyzstan, Hungry, and Turkey, which are hybrid or soft-authoritarian countries, that use digital transformation differently from purely authoritarian countries (Sipahi and Saayi, 2024; Das and Kwek, 2024; Dyussenov, 2025; Mahapatra, 2025; Perdana and Mokhtar, 2025). In Singapore, programs such as “Smart Nation” use digital technology (including AI) to enhance business productivity, promote smart city management, and foster workforce skills.

Despite the UAE and Singapore being two different countries in terms of their governing systems (the former a unitary parliamentary republic and the latter a monarchy), their approach to digital transformation is closely similar. They both employ digital transformation to advance economic and social development and to promote self-regulation among citizens (Sipahi and Saayi, 2024; Das and Kwek, 2024; Perdana and Mokhtar, 2025; Koch, 2025). The UAE, for example, employs a hybrid approach, with digital monitoring as part of state-led efforts. Data from government and private platforms support service improvement and subtle social regulation. Smart infrastructure, including traffic monitoring, biometrics, and AI-powered services, generates large social datasets. These practices are used to influence behavior, allocate resources, and encourage civic compliance (Alaleeli and Alnajjar, 2020; Tariq, 2023; van Dijck et al., 2018).

In contrast to critiques of Western digital capitalism and countries such as China, Russia, and Iran that use it to control their citizens, the UAE embeds surveillance practices within its national development objectives, encompassing economic diversification, urban planning, and cultural modernization. Though the Gulf states such as the UAE might also use digital transformation as a means of social and political discipline, the aim of this paper is to highlight how the country collaborates with the private sector (private data platforms) to use digital technology to advance its economy, promote governance, and influence everyday activities of citizens, thereby fostering citizen satisfaction with public services and fostering self-regulation among citizens. Data-driven governance in this context is often articulated as a public good, reinforcing state legitimacy while producing new forms of social inequality linked to digital access, literacy, and participation. This approach aligns with contemporary debates about the societal implications of artificial intelligence, illustrating the intersections of power, knowledge, and technology in everyday life (Warschauer, 2003; Couldry and Mejias, 2019; Eren and Çay, 2025).

### 2.4 Identity, citizenship, and digital practice in Gulf societies

The rapid adoption of digital technologies also transforms identity and citizenship in Gulf societies. Emirati and expatriate populations engage with platforms differently, creating distinct modes of digital sociality and hybrid identities (Hu and Cheong, 2021). Zayani (2015) emphasizes that social media in the Gulf fosters both networked public spaces and subtle forms of surveillance, influencing how individuals negotiate cultural norms, public reputation, and social belonging. In the UAE, digital citizenship is not merely a legal status but also a set of performative and behavioral expectations, mediated by technology and aligned with state narratives of modernity and progress (Sarker et al., 2023; Ahmat et al., 2024; Alzaabi et al., 2025).

Digital infrastructure fosters new forms of social distinction, including digital capital, algorithmic literacy, and platform-mediated visibility. These factors shape both individual and collective experiences of inclusion, opportunity, and recognition, reflecting broader sociological concerns about inequality, stratification, and the social shaping of technology (Bourdieu, 1986; Ziadah, 2024).

### 2.5 Contribution to the literature

First, this paper adds to the literature in digital sociology by extending the concept of digital habitus beyond Western liberal contexts. Much existing theory is based on experiences of market-driven digitalization. This study shows how digital dispositions also emerge in settings where digital life is closely tied to state planning, public service delivery, and national development agendas. Second, the article contributes to studies of governmentality and surveillance by showing how digital power in the UAE operates through a combination of service provision, data collection, and normative guidance. Rather than relying primarily on coercion, digital systems encourage self-regulation by embedding expectations of efficiency, responsibility, and participation into everyday routines. Third, the article contributes to Gulf and Middle East studies by offering a sociological account of digital transformation within Gulf state modernity. It treats digitalization not as an imported model but as a locally shaped process that reflects regional histories, cultural values, and demographic diversity. In doing so, the UAE is used not only as a case study but as a site for developing concepts that can inform broader debates about digital governance in non-Western and state-led contexts.

## 3 Theoretical framework

This paper proposes an integrated conceptual framework to analyze the formation of digital habitus in the United Arab Emirates. As Figure 1 illustrates, the model draws together three strands of theory—governmentality (Foucault, 1991), surveillance capitalism (Zuboff, 2019), and Gulf state modernity—to explain the structural forces that shape people's everyday digital orientations and dispositions. Each strand captures a different but complementary dimension of digital transformation in the UAE. Taken collectively, they clarify how macro-level governance logics, market-driven data extraction, and region-specific modernization agendas converge to produce patterned forms of digital practice among residents. The framework not only identifies the conditions under which digital habitus emerges but also explains how these conditions influence social outcomes, including identity expression, civic behavior, and social stratification.

### 3.1 Governmentality and digital rationalities

Governmentality provides the first layer of the framework by highlighting how state institutions structure digital participation through policies, technologies, and administrative rationalities. In the UAE, digitalization is not merely a technical process but a governing strategy that aligns public service provision with efficiency, transparency, and behavioral management (Ahmad and Khalid, 2017). Digital platforms for identity verification, mobility, health, and public communication channel citizens toward particular forms of engagement that reinforce state visions of order, responsiveness, and smart modernization (Hujran et al., 2021). These systems do more than manage information; they actively shape expectations about how individuals should move through digital environments.

By embedding normative assumptions into service design—such as immediacy, accountability, and constant connectedness—governmentality influences the early formation of digital dispositions. Individuals come to internalize routines associated with state platforms, such as regularly consulting government apps, trusting automated notifications, and perceiving digital interactions as authoritative (Hertog, 2019). These dispositions form part of the broader digital habitus.

### 3.2 Surveillance capitalism and datafication logics

The second layer involves the influence of surveillance capitalism, which highlights how commercial platforms structure digital behavior through processes of data capture, algorithmic prediction, and behavioral nudging. While government platforms in the UAE play a central role in digital life, everyday experiences are deeply intertwined with global corporate systems such as Meta, Google, and TikTok. These systems rely on extracting behavioral data to personalize content and generate predictive models that encourage engagement (Romele and Rodighiero, 2020).

This commercial layer contributes to digital habitus in several ways. It normalizes continuous sharing, shapes taste cultures, and encourages algorithmically mediated social interaction. It also differentiates experience across gender, age, class, and nationality because algorithms respond to user histories that are themselves shaped by social location. For example, affluent youth in Dubai may receive content reinforcing cosmopolitan consumption patterns, while migrant workers may be directed toward transnational communication platforms that maintain links with home countries. These patterned exposures gradually sediment into durable digital dispositions and sensibilities.

### 3.3 Gulf state modernity and regional conditions

The third layer of the framework builds on scholarship that emphasizes the specificity of Gulf state modernity—rapid development driven by state-led visions (Brzuszkiewicz, 2025), demographic diversity, and deeply rooted cultural norms. The UAE's digital landscape cannot be adequately understood without accounting for this distinctive configuration. Digital transformation is linked to national future-oriented agendas such as Vision 2021 and the broader move toward a knowledge economy (Hujran et al., 2021; Bi et al., 2025). At the same time, Emirati values regarding privacy, family respect, and public decorum influence how digital practices are interpreted and regulated.

Gulf state modernity introduces cultural expectations that shape how digital dispositions take form. For instance, the strong social emphasis on reputation, modesty, and intergenerational respect influences the boundaries of acceptable online behavior, contributing to the development of cautious, self-monitoring digital orientations (Giddens, 1991; Abokhodair et al., 2017). Meanwhile, the diverse expatriate population adds layers of hybrid digital practices shaped by cultural pluralism and transnational connectivity.

### 3.4 Digital habitus as the mediator

Digital habitus sits at the center of the framework as the mediating concept that links structural forces to observable social outcomes (see the conceptualized diagram below). It refers to the internalized digital orientations that emerge through repeated interactions with state systems, commercial platforms, and culturally embedded norms (Ristić and andKišjuhas, 2022; Romele and Rodighiero, 2020; Zayani, 2015; Romele, 2024). Digital habitus encompasses practical routines (how individuals use technology in everyday life), perceptual tendencies (how they interpret digital content), and ethical or moral considerations (how they evaluate online actions).

The concept is useful because it explains how digital behavior becomes patterned and durable without assuming rigid determinism. Individuals develop digital dispositions that reflect their social location, demographic background, and exposure to governance structures and market systems. These dispositions shape how they participate online, how they trust institutions, and how they relate to communities.

### 3.5 From digital habitus to social outcomes

The emergence of a digital habitus in the UAE gradually shapes how individuals understand themselves, relate to the state, and form connections with others. One of the most visible outcomes is the reworking of identity production (Ypma, 2009). As people move between government platforms, social media networks, and workplace technologies, they learn to present themselves in ways that align with dominant expectations of efficiency, respectability, and social responsibility. These spaces encourage a form of curated visibility—where identity is negotiated through small decisions about what to share, when to engage, and how to appear credible or trustworthy (Jurgenson, 2019). Over time, these practices become embedded dispositions that guide how individuals imagine and manage their public selves.

Digital habitus also influences civic engagement, not only through formal e-government participation but through subtler shifts in how people perceive authority and public institutions. Regular interaction with well-designed digital services can reinforce trust in state systems and normalize a sense of responsiveness and order. At the same time, algorithmic cues, notification cycles, and platform routines encourage a pattern of civic attentiveness that blends compliance with an internalized belief in collective progress. These orientations shape how people respond to public directives, interpret risk, and participate in national initiatives.

A third outcome relates to social stratification. While digital modernization in the UAE aims for inclusivity, the everyday competencies required to navigate digital systems—such as comfort with English-language interfaces, technological literacy, access to devices, or familiarity with platform cultures—can reproduce existing lines of inequality. Those who easily adapt to digital expectations may find smoother pathways to institutional services and social recognition, while others encounter subtle barriers. Digital habitus thus becomes a mechanism through which advantages accumulate more readily for some groups than for others, reinforcing or reconfiguring existing hierarchies.

Finally, community formation increasingly unfolds in hybrid digital spaces where relationships are sustained through shared platforms rather than physical proximity. Digital habitus shapes how people join groups, exchange support, and signal belonging in ways that resonate with local norms of sociability. Expatriates and citizens alike build communities through WhatsApp groups, neighborhood platforms, and professional networks, often blending cultural values with digital forms of interaction. These patterns can strengthen social cohesion, foster new forms of solidarity, or produce fragmented micro-communities that operate primarily through digital channels.

Together, these outcomes illustrate how digital habitus serves as a bridge connecting technological infrastructures to lived social realities. It becomes the medium through which digital transformation is translated into everyday identities, civic dispositions, social boundaries, and communal ties—ultimately shaping the evolving social landscape of the UAE.

### 3.6 Conceptual framework

The conceptual framework, therefore, positions digital habitus as a central analytical tool that explains how structural forces translate into lived digital experience and, ultimately, into broader social transformations in the UAE.

## 4 Operationalization

Since this article is a theoretical work that aims to develop a conceptual framework, which does not operationalize many of the concepts and variables it introduces, the researcher proposes indicators for future studies to test the concept of digital habitus. Doing so will help convert the framework's key dimensions (governmentality, surveillance capitalism, and Gulf state modernity) into empirical indicators that uncover how digital dispositions manifest in everyday life. Because habitus refers to internalized tendencies expressed through routine behavior, measuring digital habitus requires indicators that capture both observable practices and the attitudes that sustain them. In this respect, a mixed-methods approach is particularly suitable for this task. While quantitative tools such as surveys can help identify patterns of digital engagement across populations, qualitative methods (including interviews, ethnographic observation, and discourse analysis) will help explain how individuals interpret these practices and how norms surrounding digital systems become internalized. Integrating these approaches will allow researchers to examine not only how people use digital platforms, but also how they adapt their expectations and behavior to the institutional and cultural environments embedded in those systems.

To examine digital habitus through routine digital practices and competencies, indicators such as the frequency of interaction with government applications, reliance on digital identification systems, participation in online transactions, and patterns of social media engagement could be particularly useful. While these indicators are essential, attitudinal indicators are equally important because they capture the internalization of digital norms. These indicators can be operationalized through surveys, especially to examine users' trust in digital governance systems, perceptions of the efficiency of online services, and willingness to share personal data with institutions or platforms. For example, a Likert scale can be used to examine whether digital services improve efficiency or increase trust in institutions, thereby helping identify normative orientations reflecting the acceptance of digitally mediated governance.

Governmentality, another pillar of this conceptual framework, can be empirically operationalized through engagement with state-led digital infrastructures. Indicators may include how frequently individuals use government platforms, respond to automated notifications, or rely on digital channels to complete administrative tasks. Compliance behaviors (such as completing online renewals or updating official information through government applications) can also indicate the degree to which digital governance structures shape everyday routines. Future research may employ qualitative methods to complement these measures by exploring how individuals perceive the authority and legitimacy of digital state systems. To operationalize surveillance capitalism and its influence, future research may examine it through patterns of interaction with commercial platforms and attitudes toward data practices. This measurement could be carried out using indicators such as the range of platforms used, levels of content sharing, responsiveness to algorithmic recommendations, and the degree of personalized media consumption. Researchers operationalizing this pillar may also examine awareness of data collection and perceptions of digital privacy through observations of online self-presentation, influencer culture, and engagement with targeted advertising, the factors that further illustrate how platform economies shape digital behavior and symbolic status online.

Cultural norms associated with Gulf modernity and context create a third dimension of measurement. Future research may use practices related to reputation management, expectations regarding modesty in online self-presentation, and the influence of family or community norms on digital communication as indicators to examine this pillar. Given the possibility that qualitative accounts of appropriate online conduct, visibility on social media, and concerns about social reputation can reveal how global platforms are interpreted through locally embedded cultural values.

To thoroughly capture these dimensions, researchers may construct composite measures that combine behavioral, attitudinal, and cultural indicators. A Digital Habitus Index, for example, could integrate measures of digital service use, digital literacy, trust in online governance, platform participation, and perceived norms of digital conduct. Such indices would allow comparison across demographic groups—including age, nationality, education, and occupation—and help identify variations in digital dispositions within a population. Operationalizing digital habitus ultimately links theoretical analysis to empirical inquiry. By identifying measurable indicators, scholars can evaluate how digital infrastructures, governance systems, and cultural norms interact to shape everyday digital behavior. Future comparative research across Gulf societies or between different governance contexts may further clarify how distinct forms of digital habitus emerge and how digital transformation reshapes social life.

## 5 Discussion

The conceptual framework highlights that digital life in the UAE is shaped by overlapping structural forces rather than a single dominant influence. This section explains how digital habitus emerges under these conditions and its implications for identity, community, stratification, and civic behavior.

### 5.1 Identity in a structured digital environment

The dual logics of state governance and commercial platform incentives shape digital identity practices in the UAE (Ahmat et al., 2024; Alali, 2024). The state's emphasis on secure digital identity systems has normalized forms of self-presentation that privilege legitimacy, accuracy, and accountability. At the same time, social media platforms encourage creative self-representation, lifestyle visibility, and aspirational consumption. Digital habitus mediates these impulses, producing hybrid identity practices that balance cultural expectations with global digital norms.

These hybrid identities are particularly evident among Emirati youth, who navigate expectations of modesty and respect while engaging with global influencer cultures. Meanwhile, expatriate residents often adopt identity practices that maintain ties to home communities while integrating into the UAE's multicultural environment (Al-Etaibi, 2023).

### 5.2 Community and belonging in a multicultural context

Digital infrastructures in the UAE also transform the concept of community. Traditional forms of sociality—such as neighborhood majlis, family gatherings, and religious congregations—now coexist with digital communication channels that extend the reach of social networks. Platforms such as WhatsApp, government messaging systems, and social media enable the creation of virtual communities that cross spatial and social boundaries. These communities are shaped by the principles of algorithmic governance, which influence engagement through structured access, notifications, and reward systems.

This duality produces both opportunities and challenges for social cohesion. On the one hand, digital platforms enhance connectivity, facilitate information dissemination, and foster shared civic norms. On the other hand, reliance on digital infrastructures introduces asymmetries in access and participation, potentially excluding individuals with limited technological competence or resources. In effect, community engagement becomes partially mediated by digital habitus, reinforcing patterns of inclusion and exclusion in accordance with the hybrid governance model (Couldry and Mejias, 2019; Davidson, 2019). For instance, during public health campaigns or urban planning consultations, residents with strong digital habitus can participate effectively, provide feedback, and influence outcomes, while others may remain marginalized. The UAE government's emphasis on digital participation as a marker of citizenship illustrates how digital habitus functions as a conduit for both inclusion and stratification.

Digital habitus influences how individuals navigate these varied digital spaces (Annaki et al., 2025). For many Emiratis, community formation is closely tied to family and tribal networks, which extend into curated digital groups. For expatriates, digital communities often reflect transnational networks, religious affiliations, or occupational ties. The framework helps explain why community fragmentation and diversification coexist in the UAE's digital ecology.

### 5.3 Social stratification and digital inequalities

The UAE's rapid digital transformation has not only enhanced administrative efficiency but also produced new axes of social differentiation. Digital habitus often develops unevenly across social groups because individuals are differently positioned within governance systems, market platforms, and cultural expectations (Ragnedda and Gladkova, 2020). Class, nationality, gender, and occupation shape exposure to digital technologies and influence the development of digital competencies. For example, individuals (such as skilled professionals) who navigate digital platforms effectively enjoy advantages in civic participation, employment, and social recognition. In contrast, those with limited access or lower digital literacy face structural disadvantages (Warschauer, 2003).

Emirati citizens often benefit from preferential access to government programs mediated through platforms such as UAE Pass, whereas expatriate residents may encounter linguistic, technological, or bureaucratic barriers. Within expatriate communities, differences in digital habitus correlate with educational background, occupational status, and generational cohort, producing layered inequalities. This notion illustrates how the hybrid framework links governmental norms, algorithmic governance, and social stratification (shown in Figure 1): institutional and technological mechanisms reinforce social divisions, even as they facilitate what is presumed to be universal access to public services (van Dijck et al., 2018). These differences contribute to patterned stratification in digital outcomes, including differential access to information, varying capacities for online participation, and diverse experiences of algorithmic visibility.

### 5.4 Civic behavior and public trust

Digital governance in the UAE exemplifies a governmentality-driven model in which citizens and residents are encouraged to engage in self-regulation. Platforms such as DubaiNow and the UAE Federal e-Government Portal embed normative expectations regarding punctuality, compliance, and civic engagement. By providing feedback, reminders, and automated decision-making, these platforms induce a form of technologically mediated self-discipline, aligning individual behavior with state objectives (Foucault, 1991; Rose, 1999).

This digitally mediated self-regulation has several implications: (1) Behavioral standardization: Residents adjust actions to meet expectations embedded in digital interfaces, creating predictable patterns of interaction. (2) Norm internalization: Frequent engagement with e-government platforms encourages internalization of civic responsibilities as part of daily routines. (3) Algorithmic oversight: AI-driven monitoring subtly enforces compliance by flagging non-compliance or offering rewards for adherence, reflecting a blended form of surveillance capitalism and governmentality (Zuboff, 2019; van Dijck et al., 2018).

In practice, these mechanisms encourage digital civic responsibility, in which compliance and engagement serve as markers of social competence and legitimacy. For example, residents who promptly update digital health records, respond to civic notifications, or maintain accurate e-identification profiles effectively demonstrate adherence to institutionalized expectations, reinforcing social hierarchies structured by digital capital.

### 5.5 Cultural negotiation and hybrid modernity

As Figure 1 illustrates, the UAE's digital transformation is deeply embedded in cultural and religious contexts, illustrating the concept of Gulf state modernity. Unlike Western digital models driven primarily by market logic, the UAE integrates technological adoption with cultural values, national identity, and developmental priorities (Giddens, 1991; Ulrichsen, 2016). Digital habitus is thus hybrid, blending traditional social norms with contemporary technological practices. For example, online education platforms reflect both global pedagogical standards and culturally specific expectations regarding gender norms, family involvement, and Islamic ethics. Similarly, public digital campaigns around environmental sustainability or civic engagement are framed not only in terms of efficiency but also as part of national identity formation, encouraging residents to enact behaviors that reinforce collective modernization goals (Sarker et al., 2023; Ahmat et al., 2024; Alzaabi et al., 2025).

This hybridization of digital habitus ensures that modernization does not disrupt cultural continuity but rather channels it through new technological modalities. Citizens internalize a dual set of expectations: compliance with institutional digital norms and alignment with culturally embedded values, producing distinctively Gulf-specific patterns of social conduct.

## 6 Conclusion

This paper has proposed a conceptual framework that links governmentality, surveillance capitalism, and Gulf state modernity to explain the formation of digital habitus in the United Arab Emirates. By placing digital habitus at the center of the analysis, the framework shows how large-scale structures of power and technology are translated into everyday practices, orientations, and social outcomes.

The study theorizes that neither state control nor market-driven forces alone can help us understand how these forces shape digital life in the UAE. Instead, digital dispositions emerge from the interaction of governance strategies, commercial platforms, and culturally embedded ideas of progress and social order. These forces shape how people present themselves, engage with public institutions, form communities, and experience inclusion or exclusion.

Beyond the UAE, the framework has wider relevance. As digital governance expands in many parts of the world, particularly in state-led and authoritarian-modernist contexts, there is a growing need for concepts that capture how digital systems are lived and internalized in everyday life. By theorizing digital habitus in the Gulf context, this study challenges Western-centered assumptions in digital sociology and encourages more comparative, regionally focused research on digital transformation.

## Data Availability

The original contributions presented in the study are included in the article/supplementary material, further inquiries can be directed to the corresponding author/s.
